# Acute hyperoxia reveals tonic influence of peripheral chemoreceptors on systemic vascular resistance in heart failure patients

**DOI:** 10.1038/s41598-021-99159-2

**Published:** 2021-10-21

**Authors:** Stanislaw Tubek, Piotr Niewinski, Bartlomiej Paleczny, Anna Langner-Hetmanczuk, Waldemar Banasiak, Piotr Ponikowski

**Affiliations:** 1 Institute of Heart Diseases, Wroclaw Medical University, Borowska 213, 50-556 Wrocław, Poland; 2 Institute of Heart Diseases, University Hospital, Wrocław, Poland; 3 Department of Physiology, Wroclaw Medical University, Wrocław, Poland; 4 Department of Cardiology, Centre for Heart Diseases, 4th Military Hospital, Wrocław, Poland

**Keywords:** Vasodilation, Heart failure

## Abstract

Peripheral chemoreceptors’ (PCh) hyperactivity increases sympathetic tone. An augmented acute ventilatory response to hypoxia, being a marker of PCh oversensitivity, was also identified as a marker of poor prognosis in HF. However, not much is known about the tonic (chronic) influence of PCh on cardio-respiratory parameters. In our study 30 HF patients and 30 healthy individuals were exposed to 100% oxygen for 1 min during which minute ventilation and hemodynamic parameters were non-invasively recorded. Systemic vascular resistance (SVR) and mean arterial pressure (MAP) responses to acute hyperoxia differed substantially between HF and control. In HF hyperoxia caused a significant drop in SVR in early stages with subsequent normalization, while increase in SVR was observed in controls. MAP increased in controls, but remained unchanged in HF. Bilateral carotid bodies excision performed in two HF subjects changed the response to hyperoxia towards the course seen in healthy individuals. These differences may be explained by the domination of early vascular reaction to hyperoxia in HF by vasodilation due to the inhibition of augmented tonic activity of PCh. Otherwise, in healthy subjects the vasoconstrictive action of oxygen remains unopposed. The magnitude of SVR change during acute hyperoxia may be used as a novel method for tonic PCh activity assessment.

## Introduction

Myocardial injury causing heart failure with reduced ejection fraction (HFrEF) is a beginning of autonomic storm, which leads to further disease progression affecting both morbidity and mortality^1^. Numerous pathophysiological mechanisms, shifting the autonomic balance towards sympathetic domination, have been described including: decreased input from arterial baroceptors and pulmonary stretch receptors and increased signaling from renal afferents, muscle ergoreceptors, cardiac receptors and chemoreceptors^1^. Despite constant improvement in pharmacological and device therapy life expectancy in HFrEF is still poor with the 1-year all-cause mortality rate at 23.6% for acute cases and 6.4% for chronic ones^2^. Hence, the implementation of novel approaches, engaging not-yet-covered mechanisms, is necessary to improve patients’ survival.

One of the mentioned pathophysiological reflexes leading to HFrEF progression is an activation of peripheral chemoreceptors (PCh). In experimental animal models deterioration in left ventricular ejection fraction (LVEF) was clearly accompanied by an increase in peripheral chemoreceptors (PCh) sensitivity and sympathetic overactivity^3,4^. Bilateral PCh deactivation in the same models improved autonomic balance, decreased arrhythmic burden, and restrained left ventricle wall fibrosis preventing further decline in LVEF when compared to sham animals^3,4^. Moreover, early bilateral carotid body denervation improved survival in rats with induced ischemic cardiomyopathy^3^.

In humans with HFrEF, stimulation of PCh with hypoxia increases sympathetic tone—measured directly with muscle sympathetic nerve activity (MSNA)^5^. Oversensitivity of these structures, defined as exaggerated ventilatory response to transient PCh activation with hypoxia (hypoxic ventilatory response—HVR), is a well-defined independent factor for poor prognosis in HFrEF^6^. Moreover, the prevalence of PCh oversensitivity is still high in a contemporary HFrEF population in spite of optimal medical treatment^7^. Finally, PCh surgical excision significantly reduces sympathetic activity, assessed with MSNA, in pre-selected HFrEF patients^8^.

Surgical, percutaneous or pharmacological deactivation of PCh appears as a promising novel treatment for sympathetically mediated diseases such as HFrEF and arterial hypertension^8–10^. However, therapy should be tailored according to the individual pattern of reflex abnormalities engaged in the development and progression of the disease states. It could be hypothesized that only patients with increased tonic PCh input (tonicity) will benefit from the chronic PCh deactivation. Thus, it is not clear whether acute PCh activity (triggered, HVR) could serve as a reliable predictor of the favorable response. It seems that the effects of transient inhibition, rather than, the effects of transient stimulation, would better correspond with the result of procedures targeting PCh. However, so far the simple, non-invasive screening test for the augmented PCh tone is not available^11^.

Hyperoxia is one of a well-identified PCh inhibitors; however, it also exerts direct vasoconstrictive effect^12–14^. In healthy and HFrEF subjects prolonged hyperoxic exposure increases systemic vascular resistance (SVR)^13,15,16^. Opposite change—drop in SVR—was described by Sinski et al. in hypertensive subjects during the first minute of hyperoxic exposure^17^. The influence of acute hyperoxia on hemodynamics in HFrEF has not been described so far.

To further elucidate the mechanism of SVR changes and their time-dependence we designed this study in which the hemodynamic changes were assessed during one minute long exposure to 100% hyperoxia in subjects with the presumably high tonic activity of PCh (HFrEF population). These changes were then compared to healthy individuals in whom PCh tonic activity was presumed to be low. To confirm the relationship between SVR changes and the tonicity of PCh the study protocol was performed in two patients before and after bilateral carotid body resection—the procedure itself was a part of another project^8^. We hypothesize that the hyperoxia-induced drop in SVR in the early phase of the exposure is a result of the inhibition of the augmented tonic activity of PCh.

## Results

The study protocol consisted of two parts: (1) assessment of the effects of acute hyperoxia on hemodynamic and respiratory parameters; (2) evaluation of individual peripheral chemosensitivity to hypoxia (HVR) using transient hypoxia method.

### The baseline data

Parameters from one minute of baseline preceding the hyperoxic exposure (B) were averaged. LVEF, mean arterial pressure (MAP), end-tidal carbon dioxide concentration (ETCO_2_) and cardiac output (CO) were significantly higher in healthy individuals, when HVR and minute ventilation (VI) were significantly higher in HFrEF patients. There were no significant differences in body mass index (BMI), haemoglobin level, creatinine serum level, heart rate (HR), systemic vascular resistance (SVR) and blood oxygen saturation (SpO_2_) between studied groups. More details can be found in Table 1.

**Table 1:** Subjects’ demographic data and baseline parameters presented as mean ± SD.

	HFrEF n = 30	Controls n = 30	*p* value
Age [year]	62 ± 10	61 ± 10	0.92
Sex [male/female]	30/0	30/0	
BMI [kg (m^2^) ^−1^]	27.1 ± 4	27.5 ± 5	0.88
LVEF (%)	27.4 ± 7	61.2 ± 4	< 0.01
NTproBNP [pg ml^−1^]	2804 ± 2339	–	–
Hb [g%]	14.3 ± 1.4	14.6 ± 1	0.24
Creatinine [mg dl^−1^]	1.09 ± 0.26	0.99 ± 0.15	0.2
Hypertension [%]	43	–	–
Diabetes [%]	33	–	–
Peak VO_2_ [ml kg^−1^ min^−1^]	16.6 ± 5.4	–	–
HVR [l min^−1^%^−1^]	0.6 ± 0.4	0.3 ± 0.2	< 0.01
**Therapy [%]**			
B-blockers	100%	–	–
ACEI/ARB	100%	–	–
Aldosteron antagonists	90%	–	–
Loop diuretics	70%	–	–
Thiazides	70%	–	–
**Baseline parameters**			
HR [bpm]	72 ± 11	69 ± 11	0.36
MAP [mmHg]	78 ± 8	88 ± 10	< 0.01
SVR [dyn s cm^−5^]	1239 ± 380	1180 ± 317	0.64
CO [l min^−1^]	5.61 ± 1.3	6.3 ± 1.2	0.047
VI [l min^−1^]	12.1 ± 6.2	9.3 ± 2.8	< 0.01
SpO_2_ [%]	95 ± 2	96 ± 2	0.06
ETCO_2_ [mmHg]	34.2 ± 5.6	36.0 ± 3.9	0.03

Values are presented as mean ± SD.

*HFrEF* heart failure with reduced ejection fraction, *BMI* body mass index, *LVEF* left ventricle ejection fraction, *NTproBNP* N-terminal prohormone of brain natriuretic peptide, *Hb* haemoglobin level, *peak VO2* peak oxygen consumption, *HVR* individual peripheral chemosensitivity to hypoxia, *ACEI* angiotensin-converting enzyme inhibitors, *ARB* angiotensin receptor blockers, *HR* heart rate, *MAP* mean arterial pressure, *SVR* systemic vascular resistance, *CO* cardiac output, *VI* minute ventilation, *SpO*_*2*_ oxygen saturation, *ETCO*_*2*_ end tidal carbon dioxide.

### The influence of acute hyperoxia on measured parameters

Parameters from middle 20 s of the exposure (H1), last 20 s of the exposure (H2) and 20 s following the exposure (H3) were averaged and compared with B.

**Figure 1:**
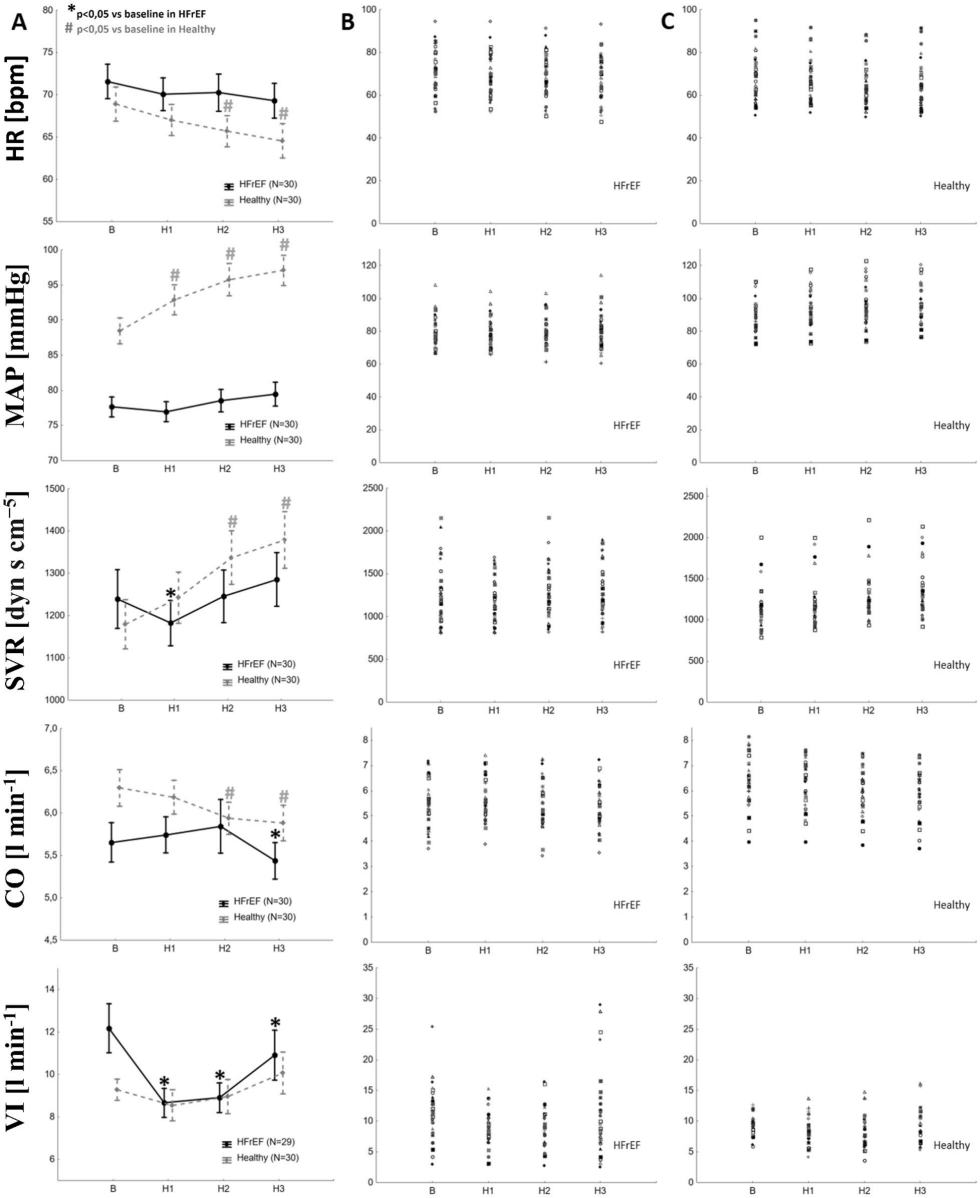
The influence of hyperoxia on measured parameters. Column A—changes in measured parameters (data presented as mean ± SEM). *p < 0.05 vs. baseline in the post-hoc analysis for HFrEF; ^#^p < 0.05 vs. baseline in the post-hoc analysis for Controls. Column B—individual data in HFrEF. Column C—individual data in Controls.

In HFrEF subjects hyperoxia caused initially a decrease in SVR (H1) with subsequent normalization of the parameter during H2 and H3. CO did not change during H1 and H2, but was reduced in H3. Significant increase in SpO_2_ and decline in VI were found during H1, H2 and H3. HR, MAP and ETCO_2_ were unchanged during studied period. More details can be found in Fig. 1. All post-hoc comparisons can be found in Table 2.

**Table 2:** The influence of hyperoxia on measured parameters. Data presented as mean ± SD.

Measured parameter	HFrEF					Controls				
	B	H1	H2	H3	*p* value	B	H1	H2	H3	*p* value
HR [bpm]	72 ± 11	70 ± 11	70 ± 12	69 ± 11	0.06	69 ± 11	67 ± 10	66 ± 10 ^a^	65 ± 11 ^ab^	< 0.01
MAP [mmHg]	78 ± 8	77 ± 8	79 ± 9	79 ± 9^b^	0.01	88 ± 10	93 ± 12^a^	96 ± 13^ab^	97 ± 12^ab^	< 0.01
SVR [dyn s cm^−5^]	1239 ± 380	1174 ± 299^a^	1246 ± 342^b^	1285 ± 346^b^	< 0.01	1180 ± 317	1242 ± 332	1337 ± 346^ab^	1379 ± 367^ab^	< 0.01
CO [l min^−1^]	5.61 ± 1.3	5.7 ± 1.1	5.8 ± 1.7	5.4 ± 1.2^ab^	< 0.01	6.3 ± 1.2	6.2 ± 1.1	5.9 ± 1^ab^	5.9 ± 1.1^ab^	< 0.01
VI [l min^−1^]	12.2 ± 6.2	8.7 ± 3.7^a^	8.9 ± 3.8^a^	11 ± 6^a^	< 0.01	9.3 ± 2.8	8.5 ± 4.1	9.0 ± 4.4	10.1 ± 5.4^b^	< 0.01
SpO_2_ [%]	95 ± 2	97 ± 3^a^	98 ± 2^ab^	98 ± 2^ab^	< 0.01	96 ± 2	98 ± 1^a^	99 ± 1^ab^	99 ± 1^a^	< 0.01
ETCO_2_ [mmHg]	34.2 ± 5.9	34.3 ± 6.1	34.0 ± 6.3	34.0 ± 5.6	0.61	36.0 ± 3.9	35.5 ± 4	35.2 ± 4.9	35.3 ± 4.5	0.07

Values are presented as mean ± SD.

*HFrEF* heart failure with reduced ejection fraction, *B* baseline, *H1* from 20 to 40 s of hyperoxia, *H2* last 20 s of the hyperoxia, *H3* 20 s following the hyperoxic exposure, *HR* heart rate, *MAP* mean arterial pressure, *SVR* systemic vascular resistance, *CO* cardiac output, *VI* minute ventilation, *SpO*_*2*_ oxygen saturation.

^a^p < 0.05 vs B, ^b^p < 0.05 vs H1, ^c^p < 0.05 vs H2.

In controls hyperoxia increased SVR and decreased CO and HR during H2 and H3. Significant rise in MAP and SpO_2_ was found during H1, H2 and H3. VI and ETCO_2_ remained unchanged during whole studied period. Look at Fig. 1 and Table 2 for all post-hoc comparisons.

### Differences in absolute changes from baseline in measured parameters between studied groups

**Figure 2:**
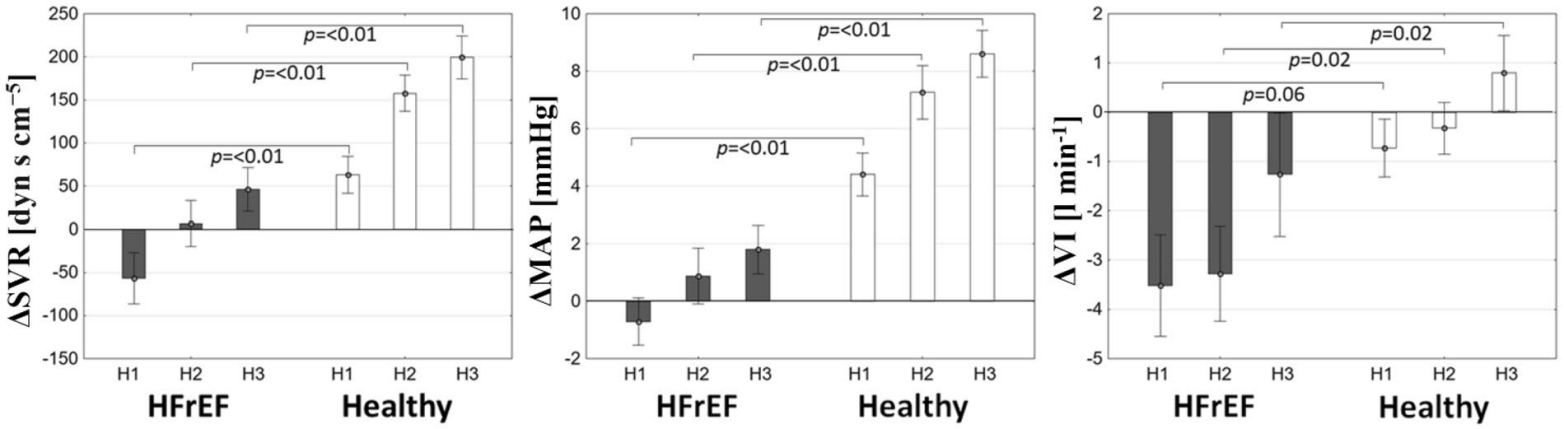
Differences in the response to hyperoxia between HFrEF and healthy subjects. Values of absolute change from baseline are presented as mean ± SEM.

SVR and MAP responses to hyperoxia differed substantially between studied populations in each time-period (Fig. 2). More profound drop in VI in HFrEF subjects was observed in H2 and H3; however, a trend towards lower VI was observed also during H1 (Fig. 2). Changes in HR, CO, ETCO_2_ and SpO2 during hyperoxic challenge did not differ between studied groups (p = NS for all).

### The influence of bilateral carotid body excision on the response to acute hyperoxia

**Figure 3:**
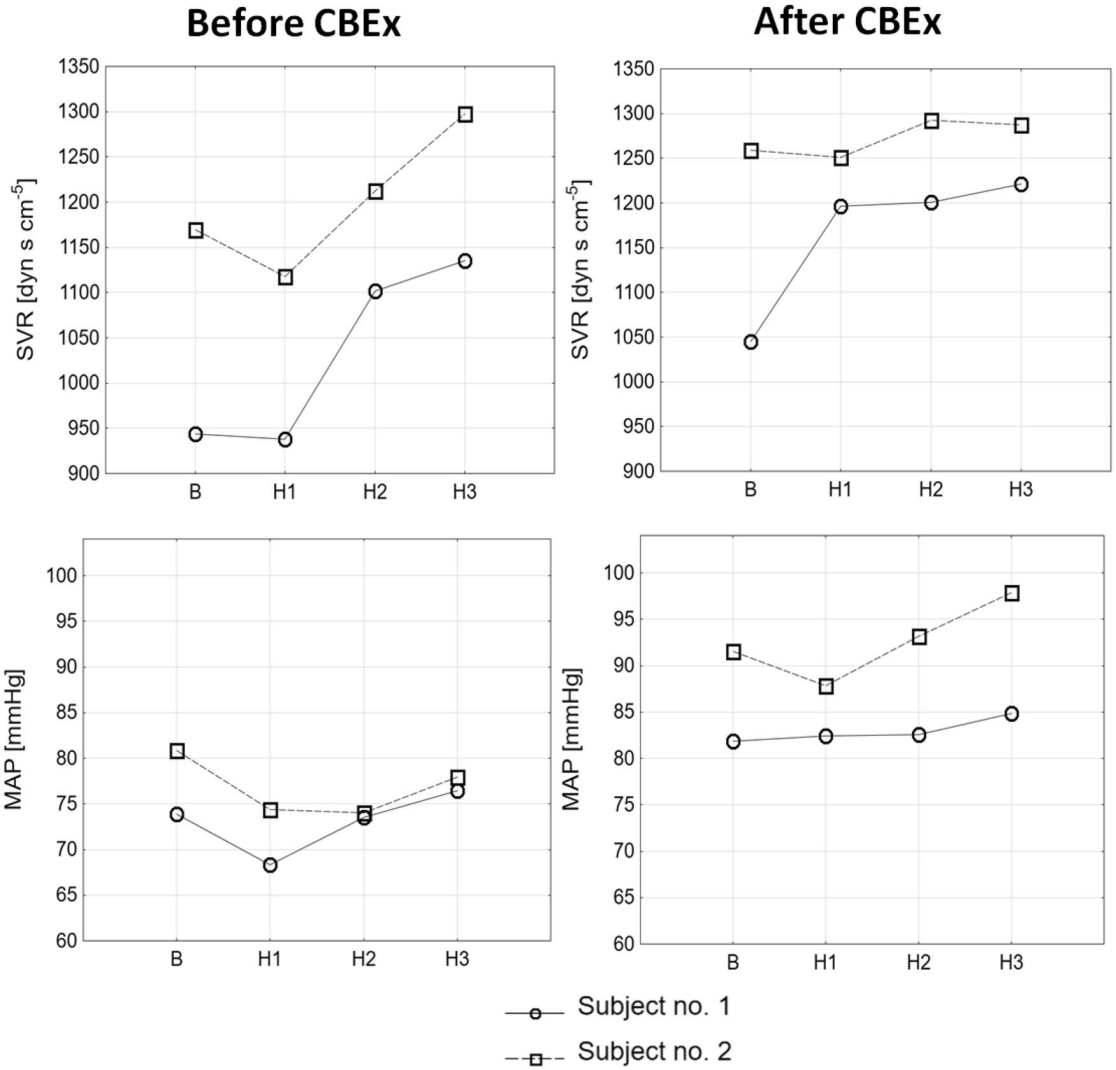
The influence of hyperoxia on measured hemodynamic parameters following acute hyperoxia in a HFrEF patients before and after bilateral carotid bodies excision (CBEx).

**Figure 4:**
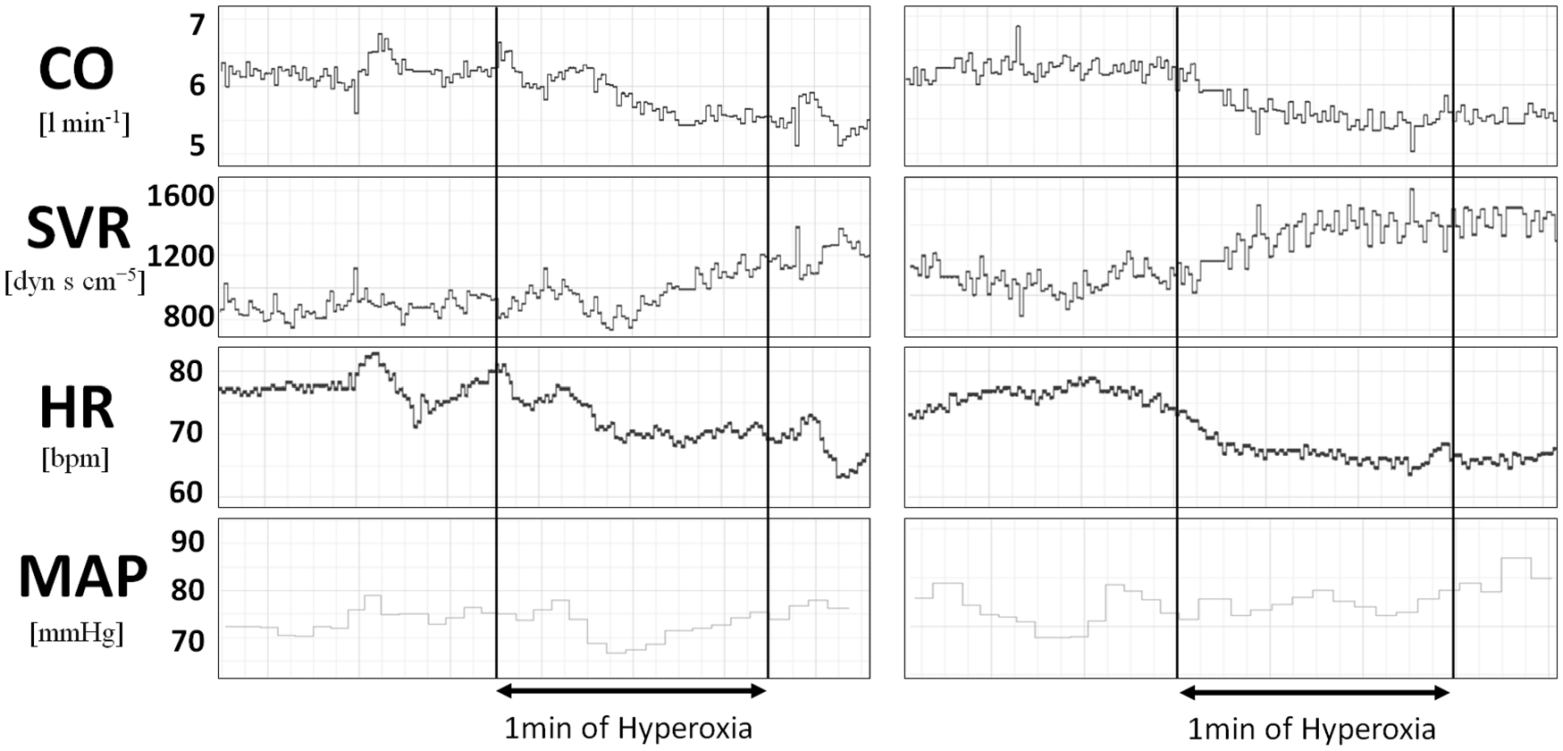
Hemodynamic response to hyperoxia before (left panel) and after bilateral carotid bodies excision (right panel) in HFrEF patient.

The changes in hemodynamic parameters in response to acute hyperoxia before and after bilateral carotid body resection are presented in Figs. 3 and 4.

### Interactions between hypoxic ventilatory response and hyperoxia-induced changes

There was no relationship between individual HVR and hyperoxia-induced changes in SVR, MAP, HR, CO and VI both in HFrEF and control groups (p = NS for all).

## Discussion

In the current paper we describe time dependent changes in hemodynamic parameters caused by acute hyperoxia in the group of HFrEF patients. The observed response differs substantially between HFrEF and healthy individuals in regards to MAP and SVR. This discrepancy is caused by hyperoxia-induced inhibition of overactive PCh in HFrEF patients, which is further supported by the restoration of the normal pattern of vasomotor response following bilateral carotid body excision.

The hemodynamic effects of sustained normobaric hyperoxia, defined as an exposure lasting from 5 to 60 min, are well described. According to the metanalysis of the 33 studies performed in humans, published by Smit et al., hyperoxia (1) decreased HR and CO; and (2) increased SVR and MAP in healthy subjects. In HFrEF patients same effects were found regarding CO and SVR, but HR and MAP remained unchanged^13^. Acute (less than a minute) hyperoxia is well known to inhibit ventilation and is used to assess PCh sensitivity (Dejours test)^18^. In the only study, which investigated the influence of hyperoxia on hemodynamics during the first minute of the exposure, Sinski et al. described drop in MAP and SVR in hypertensive but not in healthy subjects^17^. To our knowledge there are no studies describing changes of hemodynamic parameters in the first minute of hyperoxic exposure in patients with HFrEF.

From physiological point of view, at the level of microvasculature, hyperoxia alters smooth muscle cells L-type Ca^2+^ channels conduction, increases endothelin secretion (via angiotensine II mechanism), reduces the bioavailability of nitric oxide and leads to reactive oxygen species formation^19^. All these actions result in an increased tone of vascular smooth muscle cells and consequent rise in SVR. In previously cited metanalysis by Smit et al. sustained hyperoxia-induced rise in SVR was twice higher in patients with HFrEF comparing to healthy individuals, what can be explained by chronic endothelial disfunction in the former group^13,20^. Thus, this mechanism cannot be responsible for the drop of the SVR during acute hyperoxia observed in studied HFrEF population.

However, apart from local vascular actions, hyperoxia exerts effects on the autonomic nervous system. It is known to decrease sympathetic tone, measured directly with muscle sympathetic nerve activity (MSNA), during exposures lasting for several minutes in healthy, HFrEF (with anemia) and hypertensive subjects^21–23^. This phenomenon is explained by the inhibition of PCh's tonic afferent signaling into brainstem sympathetic centers, leading to the decline in efferent sympathetic activity^1^. Nevertheless, some inconsistencies may be found in the results of these trials. For example in the paper by Seals et al. breathing 100% oxygen decreased MSNA in seven healthy individuals^22^, which was not the case in the healthy control group (n = 11) as described by Sinski et al.^21^. Described inconsistences in MSNA response to hyperoxia may be a result of small sample sizes resulting in selection bias reflecting natural variability of tonic PCh activity in the studied populations. The prevalence of augmented peripheral chemoreflex sensitivity (and probably also of tonicity) is significantly different between healthy and HFrEF subjects (8% and ~ 40%, respectively)^11^. The data regarding the prevalence of PCh tonicity (as measured with hyperoxia or low-dose dopamine) is missing^11,24^.

Both physiological mechanisms—local vasoconstriction and systemic vasodilatation—influence SVR simultaneously; however, the time-course of both responses is different. In the study by Jean-Louis et al. authors assessed changes of the retinal vessels diameter during systemic hyperoxia using fundus camera and digital image analyzer and found that arterial vasoconstriction begins 25.0 ± 1.9 s after 100% oxygen administration and reaches plateau after 194.2 ± 12.4 s^25^. On the other hand direct stimulation of carotid body in vivo with adenosine caused increase in systolic blood pressure and decrease in HR with onset latency of 7.15 ± 0.48 s and 4.4 ± 0.31 s respectively^26^. Based on these data we can hypothesize that vasoconstriction (the result of local biochemical changes at the tissue level) takes more time than systemic vascular tone regulation (reflex arc via autonomic nervous system). This hypothesis may explain biphasic SVR response to hyperoxia in HFrEF patients. The primary decrease in SVR could be the result of the fast, reflex arc mediated vasodilatation due to hyperoxic inhibition of PCh. Otherwise the subsequent increase in SVR during the second phase is probably the result of delayed local vasoconstriction prevailing the primary response. The dominating role of local endothelial regulatory mechanism over autonomic regulation at microvasculature level is well known phenomenon during exercise or hypoxia (“functional sympatholysis”)^27–29^ and according to our observations similar effect seems to be in play during sustained hyperoxia. Described model also explains monophasic SVR response to hyperoxia in healthy individuals. In these subjects PCh inhibition has no discernible effect on SVR as tonic activity of PCh is negligible. The rise in SVR is simply a result of locally driven microvascular vasoconstriction, which dominates the whole response. These observations are consistent with the results reported by Sinski et al., who also observed transient drop in SVR in hypertensive (the population with expected high prevalence of increased PCh tonicity) but not in healthy subjects following short-term hyperoxic exposure^17^.

In order to prove our hypothesis we included into the study two patients referred for bilateral carotid body excision and performed the study protocol before and after the procedure. Bilateral excision of carotid bodies abolished the primary (driven by the autonomic system) response to hyperoxia with no influence on the secondary, locally-mediated vasoconstriction (Figs. 3, 4).

Taking the above into consideration, acute hyperoxic exposure provides additional information regarding the influence of PCh on systemic vascular tone. The effects of PCh inhibition are masked by local vasoconstriction increasing with the time of the exposure, thus cannot be recorded during sustained hyperoxia. Among monitored parameters shift in SVR corresponds most closely with the variations in systemic vascular tone. According to our results, a significant reduction in the SVR during H1 vs baseline was found in the HFrEF population. Individual data shows that the prevalence of PCh tonic overactivity (defined as a drop greater than mean in healthy minus 2SD) was present in 17% of the subjects with HFrEF and only in 3% of healthy subjects.

In the studied group the correlation between HVR and the hyperoxia-induced changes in SVR, MAP, HR, CO and VI was not found. This may be a result of underestimation of SVR changes due to the drop of VI in HFrEF patients or of incomplete PCh inhibition by hyperoxia^30^. However, the lack of intuitively expected relationship between triggered reflex response and tonic activity of PCh is also a possibility^31^.

There are several limitations of the study. First of all, we did not measure sympathetic tone directly thus the influence of hyperoxia on systemic sympathetic activity remains a hypothesis. Secondly, we used non-invasive, beat-to-beat method for continuous MAP, CO and SVR monitoring, which does not include central venous pressure in SVR calculation. Nevertheless, the study aimed to assess the acute changes in measured parameters during hyperoxia, therefore the absolute values of the hemodynamic indices were not crucial for the study results. Thirdly, there were important differences in pharmacotherapy between studied groups, including drugs with vasodilatory properties (Table 1). These agents might have blunted hyperoxia-induced vasoconstriction; however, the reversal of the vascular response, from constriction to dilatation (seen as a primary response in HFrEF group) could not have been significantly influenced by underlying pharmacotherapy. Fourthly, SVR could have been affected by changes in VI, via pulmonary stretch receptors reflex. Acute hyperoxia had no influence on VI in healthy, but decreased the parameter in HFrEF patients. This might have influenced measured SVR changes, leading to underestimation of the SVR drop in HFrEF population. Fifthly, blood carbon dioxide concentration is known to influence both HVR and SVR. Baseline ETCO_2_ was significantly lower in HFrEF subjects, but no significant change in the parameter was found during the study period in both groups. This may again have led to an underestimation of SVR and HVR values in the HFrEF population but should not influence SVR dynamics. Finally, the study was not placebo controlled. However, patients were unaware of the timing of hyperoxic exposure due to soundless switching between inspiratory gases of the same taste and temperature.

Deactivation of the peripheral chemoreceptors in humans by interventions targeting carotid bodies (CBD) is a promising method for restoring sympathetic balance in HFrEF and hypertension. The results of the first in man studies revealed that: (1) in subjects with HFrEF and augmented HVR bilateral CBD decreased sympathetic activity and elongated exercise time^8^ and (2) in hypertensives unilateral CBD decreased blood pressure and sympathetic tone in 8 of 15 subjects with resistant arterial hypertension^9^. Precise subjects selection is necessary to increase the efficacy and safety of the CBD. Acute hyperoxic exposure with SVR assessment may play a role of a simple screening test; however, it should be validated in the future studies.

## Methods

### Studied population

The study was approved by local Ethics Committee (Komisja Bioetyczna, Wroclaw Medical University) and was performed in accordance with the latest review of the Helsinki Declaration. All subjects gave an informed written consent. Thirty HFrEF patients and 30 age- and BMI-matched healthy individuals (control group) were enrolled into the study. Additionally two patients with HFrEF referred for bilateral carotid bodies excision as a part of another project, were tested using study protocol (as described below) before and after the procedure^8^. All patients remained clinically stable and all HFrEF subjects received optimal medical treatment according to the current guidelines at least 1 month prior to the study entry^32^. Subjects excluded from study participation included those with known significant carotid artery stenosis, paced heart rhythm, suffering from significant pulmonary disease (Tiffeneau index < 70%), left ventricular ejection fraction < 15% or ≥ 40% (for HFrEF group), NYHA functional class IV, end-stage renal failure, known history of obstructive sleep apnea and a history of an acute coronary event, coronary revascularization, or stroke within 3 months preceding the study. Study participants were asked to avoid caffeine intake 24 h and nicotine 12 h before the tests. Detailed information regarding subjects’ demographic data and baseline parameters are shown in Table 1.

### Assessment of the effects of acute hyperoxia

After period of familiarization with the study apparatus (of approximately 10 min) when recording did not take place, subjects started breathing room air for 5 min. Next, they were silently switched to breathing with 100% oxygen for the following 60 s. We deliberately limited duration of the hyperoxic exposure being aware of the systemic and central actions of oxygen (including hyperoxic hyperventilation) which appear after this time^33^. Following the hyperoxic exposure subjects were allowed to rest until measured parameters had returned to the baseline levels. Parameters from one minute of baseline preceding the hyperoxic exposure (B), middle 20 s of the exposure (H1), last 20 s of the exposure (H2) and 20 s following the exposure (H3) were averaged. The parameters from the first 20 s of the hyperoxic survey (H0) were discarded, as during this phase oxygen concentrations in the breathing circuit and lungs were equalizing. The averaged values of the parameters recorded during B were compared with the parameters from H1, H2 and H3 respectively. We decided to use 20 s epochs in order to precisely characterize the time dependency of hyperoxic response.

### Assessment of individual peripheral chemosensitivity to hypoxia

We employed well established method for the assessment of PCh sensitivity to intermittent hypoxia (the hypoxic ventilatory response, HVR)^34^. Subjects breathing room air were silently switched to 100% nitrogen for 10–35 s, which caused falls in SpO_2_ to 90–65%. Hypoxic exposures, of randomized lengths, were repeated 5–8 times per test. After each administration of nitrogen subjects were allowed to rest until measured parameters had returned to baseline levels. Each ventilatory response was calculated as an average from the three largest consecutive breaths following the nitrogen administration. HVR was expressed as the slope of the linear regression describing the relationship between the single ventilatory responses and the associated nadirs of SpO_2_, including the baseline values of VI and SpO_2_.

### Measurements

Subjects were examined in the supine position, in a quiet room, using a one-way open breathing circuit with remotely controlled mechanical valve attached to the inspiratory arm, which allowed for silent switching between room air, 100% oxygen or 100% nitrogen. The expiratory arm was connected via a 1000 L min^−1^ flowhead (MLT3000L, ADInstruments) to a differential pressure transducer (FE141 Spirometer, ADInstruments) for the assessment of VI. Measured Hemodynamic parameters included: HR, CO, SVR and MAP, which were recorded non-invasively, beat-by-beat using Nexfin Finapres technique (Nexfin BMEYE B.V.). Monitor was appropriately calibrated before the test using physiological calibration. SpO_2_ was evaluated using a pulse oximeter (Radical-7, Masimo Corporation Irvine) with an ear clip. SpO_2_ recording was shifted backward by 15 s to compensate the circulatory delay. ETCO_2_ was monitored with a CO_2_ analyzer attached to the expiratory arm of the circuit (CapStar 100, CWE).

### Data and statistical analysis

Statistica 12 (StatSoft Inc.), LabChart 8 (ADInstruments), MATLAB (MathWorks) were used to analyze the data. The distribution of the variables was tested using Shapiro–Wilk’s W test. Normal distribution was found for age, haemoglobin level, baseline CO, H1 CO, H1 HR, H2 HR, H3 HR, H3 MAP; change in MAP, HR between H1 and baseline; change in SpO_2_, MAP, between H2 and baseline; for change in MAP, HR, CO, SpO_2_ between H3 and baseline; all other variables were non-normally distributed. Friedman’s rank test was employed to assess the effects of hyperoxia on measured parameters in subsequent epochs (B, H1, H2 and H3) for HFrEF and controls separately. Dunn’s test was used for post-hoc analysis. Statistical testing for differences in demographic data, baseline parameters, HVR and changes in measured ventilatory and hemodynamic parameters between HFrEF and controls were performed with unpaired Student's t-test or Mann–Whitney U test, where appropriate. The correlations between variables were assessed with Pearson correlation coefficient or Spearman’s rank correlation coefficient, where appropriate, followed by visual graph evaluation for potential non-linear relationships. Data are presented as mean and standard deviation (SD) or mean and standard error of the mean (SEM). *P* value < 0.05 was considered statistically significant.
